# Protective factors in patients aged over 65 with stroke treated by physiotherapy, showing cognitive impairment, in the Valencia Community. Protection Study in Older People (EPACV)

**DOI:** 10.1186/1471-2377-12-118

**Published:** 2012-10-05

**Authors:** Vicente Gil-Guillen, Concepcion Carratala-Munuera, Juan Carlos Andres Ortega, Domingo Orozco-Beltran, José Martínez Ribera, Avelino Pereira Expósito, Pablo Martínez Cánovas, Eduardo Payá Mora, Emilio Mayoral Azofra, Antonio Fernández Giménez, Adriana Lopez-Pineda, Patricia Lorca-Amorrich, Carlos Plaza-Sirvent, Elisabet Berzosa Sola, Ramon Salas-Rico, Angel Fernandez-Garcia

**Affiliations:** 1 Departamento Medicina Clínica, Universidad Miguel Hernández de Elche, Ctra. Nnal. 332 Alicante – Valencia s/n, Sant Joan d’Alacant, Alicante, 03550, Spain; 2 Servicio de Rehabilitación, Hospital General de Elda, Ctra. Sax-Elda, s/n, Elda, Alicante, 03600, Spain; 3 Unidad de docencia e investigación, Hospital Universitario de Sant Joan d’ Alacant, Ctra. Nnal. 332 Alicante – Valencia s/n, Sant Joan d’Alacant, Alicante, 03550, Spain; 4 Unidad de Investigación, Hospital General de Elda, Ctra. Sax-Elda, s/n, Elda, Alicante, 03600, Spain; 5 Servicio de Cardiología, Hospital General de Elda, Ctra. Sax-Elda, s/n, Elda, Alicante, 03600, Spain; 6 Servicio de Biblioteca, Hospital General de Elda, Ctra. Sax-Elda, s/n, Elda, Alicante, 03600, Spain

## Abstract

**Background:**

Family function may have an influence on the mental health deterioration of the caregivers of dependent family members and it could have a varying importance on the care of dependents. Little attention has been paid to the preparation of minor stroke survivors for the recovery trajectory or the spouse for the caregiving role. Therefore, this study protocol intends to analyze the influence of family function on the protection of patients with stroke sequels needing physiotherapy in the family environment.

**Methods/Design:**

This is an analytical observational design, prospective cohort study and using a qualitative methodology by means of data collected in the “interviews of life”. The study will be carried out by the Rehabilitation Service at Hospital of Elda in the Valencia Community.

All patients that have been diagnosed with stroke and need physiotherapy treatment, having a dependency grade assigned and consent to participate in the study, will undergo a monitoring of one year in order to assess the predictive factors depending on the dependence of the people affected.

**Discussion:**

Our research aims to analyze the perception of caregivers, their difficulties to work, and the influence of family function. Moreover, it aims to register the perception of the patients with stroke sequel over the care received and whether they feel protected in their family environment.

## Background

Globally, cerebrovascular disease impacts the lives of over 15 million people annually. It is the leading cause of adult disability and the second leading cause of death [1]. Cerebrovascular disease occurs predominantly in mid-aged and older adults. The risk of stroke, a major cerebrovascular disease category, doubles every 10 years after the age of 55 with most strokes occurring in people over the age of 65 years. Following a sentinel stroke, there is a 20% chance of having another stroke within 2 years [2]. The World Health Organization (WHO) has estimated that of those people worldwide who suffer a stroke annually, 5 million will die and another 5 million will be left permanently disabled; placing a major burden on families and communities. Although advances in stroke prevention and treatment have led to a steady decline in major stroke, the incidence and prevalence of minor stroke are increasing [3]. What is not known is how a minor stroke contributes to the burden of care and the implications this seemingly non-disabling event may have for adjustments occurring during the normal ageing process. Patients experiencing minor stroke are most often discharged directly to their home environment, with or without outpatient rehabilitation [4]. Stroke caregivers and patients are dropped into the caregiver and recipient roles acutely following the stroke event, unlike other chronic illnesses in which the caregiving role is developed over time. Little attention has been paid to the preparation of minor stroke survivors for the recovery trajectory or the spouse for the caregiving role [5].

These sudden shifts in roles and functions may further influence stroke recovery, especially when coupled with changes inherent within normal transitions associated with the ageing process [6]. This involves a physical and mental decline which affects the functional capacity and, therefore, a large number of people require daily care [7-11].

We define as dependency the permanent state in which people find themselves that, for reasons related to age, illness or disability, and linked to the lack or loss of physical, mental, intellectual or sensory impairment, require the attention of others, or significant support for Daily Life Activities or, in the case of people with intellectual disabilities or mental illness, other support for personal autonomy [12].

Much of the care for dependent people is provided informally by informal caregivers, most notably it is provided by family who is the main healthcare provider [13,14]. Informal caregivers help to keep people in their social environment, to reduce the use of formal resources and to delay or to avoid institutionalization [15,16].

The presence of a person, who requires daily care, creates a new family situation which may cause major changes in family structure and behavior patterns of its members. These changes can produce crises that threaten the stability of the family, and will affect all its members, especially the primary informal caregiver. Informal primary caregiver is defined as the family member who supports most of the physical and emotional caregiver burden [17,18]. This person is responsible to assist in basic and instrumental needs of the patient’s daily life for most of the day, without receiving financial compensation [19-21].

Other studies confirm that caregivers experience serious conflicts and problems in their relationship with members of their families, and these problems are derived from the way they understand the disease and the strategies employed to manage the situation [22,23]. The family function may have more or less influence on the deterioration of the mental health of caregivers of dependent family members, this factor has not been studied in Primary Care and it is important to know to what extent a functional family (families in which the roles of all members are set without critical points and weaknesses with no primacy positions, artificial or assumed by any member, and in which everyone works together, and contributes and cooperates equally and with enthusiasm for the collective welfare) can be a factor protective for caregiver’s health [24].

### Justification

Our research aims to assess the influence of family function in the protection of people with stroke sequelae and need for physiotherapy, in their family environment. We would like to identify, by carring up a 12-months follow-up, if a relationship between family protection and dependance grade exists. This information may help the physiotherapy team and medical team when they wish to know the prediction of the relationship between family function and patient protection.

## Methods/Design

### Main objective

To determinate the relationship between the family function and the grade of protection* of people suffering from the effects of a stroke, needed of physiotherapy treatment.

### Specific objectives

1. To identify the variables that allow us to predict the caregiver burden of patients over 65 years with stroke admitted to a physiotherapy treatment for a year.

2. To identify the dependence grade and cognitive deterioration in patients over 65 years with stroke.

3. To quantify the caregiver burden of dependent patients suffering from the effects of a stroke.

4. To know the perception of patients suffering from the consequences of a stroke, about the care they receive and if they feel protected *(their selves) in their family environment.

5. To know the perception of the caregivers of dependent patients suffering from the effects of a stroke.

* (Protection definition: “The set of actions, aid, self help and formal and informal care available to a patient in their family environment or household to reduce the chances of physical complications, mental and/or social”).

### Study design

This is an analytical observational design, prospective cohort study [25] and using a qualitative methodology by means of data collected in the “interviews of life”.

The study will be carried out with a one year follow-up from 01.01.2012 to 01.01.2013 in order to identify the predictive factors depending on the dependence grade of the patients and the caregiver burden. In order to understand whether there is a relationship between the care received and the care provided by main caregivers, qualitative research techniques will be used, through personal interviews (patient and main caregiver).

### Setting

Specialized Care Unit (Rehabilitation Service). Elda Health Department, Autonomous Community of Valencia.

### Study population

Patients diagnosed with stroke, with a dependence grade according to the Dependency Law in force in Spain [12] and needing physiotherapy treatment. These variables are based on the characteristics of the patients over 65 years, their families and the health services they receive.

Selection criteria to include patients in the study:

A. **Inclusion**:

– Age 65 years or older

– Patients diagnosed with stroke with motor effects

– Patients attend the Rehabilitation Service of Elda Hospital

– Patients with caregiver who provide most of the assistance of their care

– Informed consent

B. **Exclusion**:

– Do not agree to participate in the study.

– Do not sign the informed consent.

– Institutionalized people.

– Motor effects affecting speech (total aphasia).

– Patients without caregiver and patients with dementia.

### Data collection methods

#### Sampling method: Non probabilistic. Consecutive

The sample will be recruited from the Rehabilitation Service of Elda Hospital up to reaching the previously calculated sample size. Study will be carried out according the common clinical practice.

#### Sample size

The sample size is calculated by accepting an expected proportion of 10% patients with stroke, with a precision (i) <5% and confidence level (1-alpha) of 95%, obtaining a N = 138 patients. Assuming a 10% of loses, we estimate that N = 153 patients will be needed. So finally, 153 patients and their main caregivers will be recruited.

### Measurements

The variables will be collected interviewing patients and their caregivers or family members, by a psychologist and a physiotherapist trained for applying the questionnaires (Table 1).

**Table 1:** Study variables

**Variables**	**Collection method**
Protection	Quality measurement
Cognitive deterioration	Pfeiffer Test
AVD (Daily Life Activities)	Barthel Test
Family function	APGAR Test
Falls	WHO Questionnaire for the study of falls
Comorbidity	Charlson Comorbidity Index
Socio-demographic	Clinical record
Caregiver burden	Conversational interview
	Zarit scale test

#### Patient variables

* Protection (main variable): will be calculated using a quality measurement and will be divided in 4 categories/rates:

– Category 1: good

– Category 2: acceptable

– Category 3: regular

– Category 4: bad

* Cognitive deterioration: will be determined by Pfeiffer Test (validated in Spain), which yields scores ranging from 0 to 10, with 10 representing the best performance [26].

* AVD (Daily Life Activities): will be calculated using the Barthel Test (validated in Spain). The Barthel Index consists of 10 items that measure a person’s daily functioning specifically the activities of daily living and mobility. The items include feeding, moving from wheelchair to bed and return, grooming, transferring to and from a toilet, bathing, walking on level surface, going up and down stairs, dressing, continence of bowels and bladder. Item scores are summed to generate a total score (0 = minimum independence; 20 = maximum independence) [27].

* Falls: will be calculated using the WHO Questionnaire for the study of falls [28].

* Comorbidity: will be measured by the Charlson Comorbidity Index. It predicts the ten-year mortality for a patient who may have a range of co-morbid conditions such as heart disease, AIDS, or cancer (a total of 22 conditions). Each condition is assigned with a score of 1, 2, 3 or 6 depending on the risk of dying associated with this condition. Then the scores are summed up and given a total score which predicts mortality.

* Socio-demographic variables:

– Age: categorical measurement [29]

▪ Category 1: 65 to 70 years old

▪ Category 2: 71 to 75 years old

▪ Category 3: > 75 years old

– Sex: male or female

– Academic level of education: No education, primary studies, secondary studies, University Degree.

– Family situation: alone or not alone (3 categories)

▪ Category 1: spouse

▪ Category 2: sons

▪ Category 3: remunerated external caregivers

#### Caregiver variables

* Socio-demographic variables: age, sex, academic level of education, marital status, occupation, relationship with the patient.

* Family function: will be evaluated with the Family APGAR Questionnaire (Adaptation Partnership Growth Affection and Resolve) validated in Spain. This questionnaire rates satisfaction with family relations and distinguish five components of the family function: adaptability, partnership, growth, affection and resolve. It consists of the five questions, with three possible answers: 0 (“hardly ever”), 1 (“sometimes”), 2 (“always”). The total score range varies from 0 to 10, meaning the higher total score, the better family functioning. A global score of 7 points or more indicates family functionality, while a score of less than 7 points indicates family dysfunction [30].

* Caregiver burden: will be measured with the Zarit Burden Inventory.The Short Zarit Interview used in palliative care cases to determine the family giving up has an S of 100%, Sp 90.5%, PPV 95.45%, and NPV 100% in defining caregivers’ burden in primary care [31].

### Follow-up

Patients diagnosed with stroke (study population) and their main caregivers (study factor or exposure) will be recruited. A 12-months follow-up will be carried out on patients with stroke in order to analyze all study variables and to know the relationship between those which are predictive of the effect (when the dependence grade is higher and the family function is lower, we will observe less protection for the patient and the main caregiver).

The questionnaires will be used at the beginning of the study in order to identify the caregiver and to measure the family function, the dependence grade and the cognitive deterioration. Subsequently, the questionnaires will be passed out after 6 months and, lately, after 12 months. At the end of the 12-months follow-up, we will be able to estimate if a correlation between caregiver burden and family function with dependence grade exists, and also, to detect the protection level during the 12-months period.

### Statistical analysis

After a data quality control performed by the Research Unit of the Department of Elda Hospital (Alicante), the data will be analyzed, after 6 months and one year monitoring, using a statistical method depending on the variable rate and objectives. The final analysis of the study will be completed after the one year monitoring. It will take into account the specific probability of protection recurrence depending on the factors included in the model, including the possibility of having an accident, and whether it has a high predictive level. As association measurements the relative risk and confidence limits at 95% will be used. SPSS-PC for Windows version 15 will be used.

#### Qualitative data collection

A conversational interview [32] will be carried out using a script developed for this study with open questions to the patient. Also will be used the Zarit scale test-validated about the caregiver burden and a conversational interview to all the caregivers.

The following decisions will be included in the interview design:

a) The interviewee selection in order to provide relevant information.

b) The interviewer selection (who has the best relationship with the interviewee).

c) The most appropriate time and place for the interview to take place.

The duration of the interview will be up to 90 minutes, or until the information is saturated (when the person doesn’t provide new information or it’s repeated). The interviews will be performed by a researcher at the patient’s home or in the place the patient chooses. The interviews will be held with both the patient and caregiver separately in order to maintain their privacy so they can answer freely.

To obtain an adequate sample size in the qualitative researching the intentional non- probabilistic sampling is used: the most representative people of the phenomena under study and who meet the inclusion criteria.

Numeric samples are not used in the “qualitative research”, obtaining information from speeches that are adequate to meet the problem. Therefore, a representative sampling is used, it will be chosen depending on the variable “Family situation”, which has 3 categories. Five representative people each category, thus 15 - 20 subjects will be an adequate sample size.

The interview will be recorded previous consent of the patient and caregiver. They will be informed about the conditions of privacy (anonymity) and about the option to read the interview once finished in order to verify what is written and that it is reflective of what they have discussed.

Non-verbal language will be taken also into account.

Using a triangular methodology, we will compare this information with that obtained through quantitative methodology.

**Figure 1:**
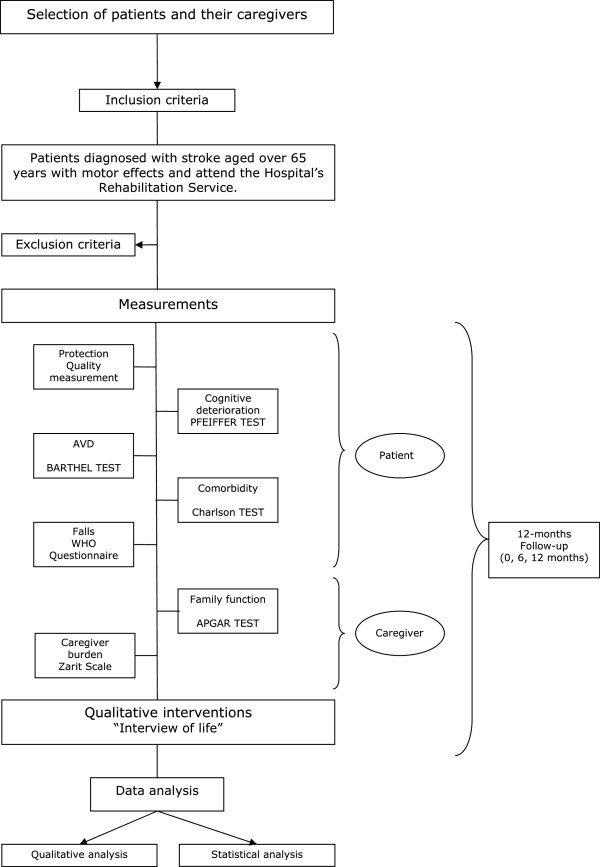
Flow diagram of the study.

An overview of study protocol is given in Figure 1.

#### Study limitations and difficulties

As for the monitoring of patients in the study, it has well defined the origin of the study subjects. The problem of possible withdrawals is controlled by the sample size and the fact that they are patients who come promptly at Physiotherapy sessions.

The conversational interview [31] shares the advantages and limits of any qualitative study. One of the most important advantages is that the participation of the interviewer and interviewed has “explicit expectations”: one for talking and the other one for listening”, the interviewer encourages the interviewee to talk constantly without contradicting. The interview based on a Patton script 1990, characterized by the preparation of a script with topics covered and be free to order the interviewer and ask questions along the match interview. The open style of this data collection technique allows obtaining a wealth of information (intensive holistic or contextual in nature) and approaches of the respondents. A lack of group interaction would be a disadvantage, but for this study to create focus groups would be more a drawback than an advantage because often moving caregivers to meet with the patient was a problem due to the lack of time, therefore a conversational interview design was decided.

### Ethical and legal issues

This study protocol has been reviewed and approved by the Ethics Committee for Clinical Trials from Elda Hospital (*Comite Ético de Investigación Clínica (CEIC) del Hospital General de Elda*), on October 25th, 2010.

The study is conducted according to the standards of the International Guidelines for Ethical Review of Epidemiological Studies (Council for International Organizations of Medical Sciences- CIOMS-Geneva, 1991) and the recommendations of the Spanish Society of Epidemiology about the review of ethical aspects of epidemiological research.

#### Confidentiality of the data

All information relative to the patient’s identity is considered confidential. The data generated during the study will be handled according to the Law 5/1999 and corresponding normative. Any researcher with access to the data used in the study will be required to sign a document guaranteeing confidentiality.

#### Informed consent

All patients must read the “Patient Information Form” and sign a document giving their consent.

## Discussion

Stroke caregivers and patients are dropped into the caregiver and recipient roles acutely following the stroke event, unlike other chronic illnesses in which the caregiving role is developed over time. Little attention has been paid to the preparation of minor stroke survivors for the recovery trajectory or the spouse for the caregiving role [5].

It has been shown by other studies that caregivers have conflicts and relationship problems with other family members. These problems arise from the way they understand the disease and set up the strategies to handle the situation [22,23]. The influence of family function could have a varying importance in the care of dependents, not having been studied in primary care [24].

There are other studies describing the prevalence and determinants of dependence associated with informal care over the health services used. However, none of these studies are focused from the perspective of the patient with stroke; all of them were carried out from the perspective of caregivers.

Our research aims to assess the influence of family function in the protection of people with stroke sequelae and need for physiotherapy, in their family environment. On the other hand, it wants to know the perception of the caregivers, their difficulties with work, and the influence of the family function. Furthermore, it aims to record the perception of the patients with stroke sequelae about the care received and whether they feel protected in their family environment.

Using a triangular methodology, we will analyze the hypothesis that “the higher dependence grade and the lower family function, the less “protection” will be observed for both patient and primary caregiver”.

An application of this study is the assessment, through the objectives, of predictive factors in terms of dependence of people affected by stroke to better understand if there is a relationship between the care received and the care provided by caregivers. Therefore, the results of our study should be of great interest for the community of physiotherapists and most relevant for the families affected.

## Abbreviations

CEIC: Comité Etico de Investigación Clínica (Ethics Committee for Clinical Trials); WHO: World Health Organization; AVD: Actividades de la vida diaria (Daily Life Activities).

## Competing interests

The authors declare they have no competing interests.

## Authors’ contributions

Conception of the idea for the study: JCAO, MCCM and VFGG. Development of the protocol, organization and funding: VFGG, JCAO, MCCM, DOB, JMR, APE, PMC, EPM, EMA, AFG and JMPR. Writing of the manuscript: JCAO,MCCM, ALP, PLA, CPS, EBS, RSR. All authors have critically read the final manuscript draft, to make contributions, and have approved the final version.

## Pre-publication history

The pre-publication history for this paper can be accessed here:

http://www.biomedcentral.com/1471-2377/12/118/prepub
